# S254. IMPLEMENTATION OF A PROGRAM FOR EARLY INTERVENTION IN PSYCHOSIS ONSET: THE EXPERIENCE OF REGIONE EMILIA ROMAGNA, NORTHERN ITALY

**DOI:** 10.1093/schbul/sby018.1041

**Published:** 2018-04-01

**Authors:** Maria Ferrara, Sinan Guloksuz, Shadie Burke, Flavia Baccari, Manuela Miselli, Alessio Saponaro, Mila Ferri, Vinod Srihari, Fabrizio Starace, Gruppo Regionale Esordi Psicotici

**Affiliations:** 1 Yale University, AUSL Modena; 2 Academic Hospital Maastricht, Yale University; 3 AUSL Modena; 4 Servizio Assistenza Territoriale, Area Salute Mentale e Dipendenze Patologiche, Regione Emilia-Romagna

## Abstract

**Background:**

Early interventions services (EIS) for psychosis are not uniformly available in the Italian public mental health care system. In 2012, Region Emilia Romagna funded the implementation of a comprehensive population based program to deliver EIS. These services provide a package of care including psychiatric consultation, family psychoeducation, case management, recovery oriented activities (e.g. supported employment, social inclusion), and physical health monitoring, consistent with international models but embedded within community mental health services (CMHS). We report feasibility, descriptors of enrolled samples, and clinical variables associated with remission.

**Methods:**

Demographic and clinical data of CMHS users that accepted EIS from January 1st, 2013 to December 31st, 2016 were acquired from paper and electronic health records in each province. Inclusion criteria were: residence in Regione Emilia Romagna, age 18–35, presence of non-organic, affective and non-affective psychotic symptoms within two years of onset. Exclusion criteria included severe intellectual disability and non-fluency in Italian. Remission was defined as a total score of 8 on the Health of Nation Outcome Scale (HoNOS) at 6 months after enrollment.

**Results:**

Six hundred and eighty-nine patients accepted EIS. Median age was 22, 93% had diagnoses of non-affective psychosis, whereas 7% affective psychosis, with a median duration of untreated psychosis (DUP) of 6 months [IQR=10; 0–120], 41% had comorbid substance use disorders, 31.1% had personality disorders, and 39% had a previous hospitalization. The proportion of migrants (23%) was almost twice that of the entire Region (11.9%). Psychiatric visits represented 44% of total utilization, whereas only 14% received at least one case management visit, 79% a family session, 19% a recovery oriented activity, and 1% physical health monitoring.

Of the sample, 460 subjects (67%) improved as presented with significant reduction in the 4 subscales scores of the follow up HoNOS, and 164 (35.7%) showed remission. Shorter DUP and lower HoNOS scores at baseline were associated with an increased likelihood of achieving remission (OR=1.03, p=0.0068, and OR=1.04, p=<0.0001, respectively), whereas the presence of personality disorder was associated with a reduced likelihood of remission (OR=0.48, p=0.0057).

**Discussion:**

EIS was acceptable to most eligible patients in regional CMHS. EIS enrollees evidenced significant clinical improvement in the first 6 months. Only a minority was diagnosed with bipolar disorder, suggesting a possible later onset of affective psychosis and reduced chance of accessing the Program.

The correlation of comorbid personality disorder with worse outcomes, suggests the need to develop a targeted treatment. The EIS were also well accepted by the high proportion of migrants. Further work is required to understand possible social determinants of psychosis onset and pathways to care in these fragile communities. The high rate of concomitant substance use at intake must be considered for developing specific pharmacological and psychoeducational treatment.

One in five patients needed admission to the inpatient unit in the first six months after onset, showing high levels of symptomatic distress. Moreover, referrals from hospital units show also possible barriers to access outpatient mental health facilities when users present with acute and urgent clinical conditions.

This report establishes the feasibility of a regional network of EIS in Northern Italy with shared data elements that will lead to useful comparisons across EIS sites within the region, and also collaborative efforts to address specific gaps in access or outcomes.

